# NVP-AUY922: a small molecule HSP90 inhibitor with potent antitumor activity in preclinical breast cancer models

**DOI:** 10.1186/bcr1996

**Published:** 2008-04-22

**Authors:** Michael Rugaard Jensen, Joseph Schoepfer, Thomas Radimerski, Andrew Massey, Chantale T Guy, Josef Brueggen, Cornelia Quadt, Alan Buckler, Robert Cozens, Martin J Drysdale, Carlos Garcia-Echeverria, Patrick Chène

**Affiliations:** 1 Novartis Institutes for BioMedical Research, Oncology Research, Klybeckstrasse 141, CH-4057 Basel, Switzerland; 2 Vernalis Ltd, Granta Park, Great Abington, Cambridge, CB1 6GB, UK; 3 Novartis Institutes for BioMedical Research, 500 Technology Square, Cambridge, MA 02139, USA; 4 Novartis Pharma AG, Forum 1, Novartis Campus, CH-4056 Basel, Switzerland

## Abstract

**Introduction:**

Heat shock protein 90 (HSP90) is a key component of a multichaperone complex involved in the post-translational folding of a large number of client proteins, many of which play essential roles in tumorigenesis. HSP90 has emerged in recent years as a promising new target for anticancer therapies.

**Methods:**

The concentrations of the HSP90 inhibitor NVP-AUY922 required to reduce cell numbers by 50% (GI_50 _values) were established in a panel of breast cancer cell lines and patient-derived human breast tumors. To investigate the properties of the compound *in vivo*, the pharmacokinetic profile, antitumor effect, and dose regimen were established in a BT-474 breast cancer xenograft model. The effect on HSP90-p23 complexes, client protein degradation, and heat shock response was investigated in cell culture and breast cancer xenografts by immunohistochemistry, Western blot analysis, and immunoprecipitation.

**Results:**

We show that the novel small molecule HSP90 inhibitor NVP-AUY922 potently inhibits the proliferation of human breast cancer cell lines with GI_50 _values in the range of 3 to 126 nM. NVP-AUY922 induced proliferative inhibition concurrent with HSP70 upregulation and client protein depletion – hallmarks of HSP90 inhibition. Intravenous acute administration of NVP-AUY922 to athymic mice (30 mg/kg) bearing subcutaneous BT-474 breast tumors resulted in drug levels in excess of 1,000 times the cellular GI_50 _value for about 2 days. Significant growth inhibition and good tolerability were observed when the compound was administered once per week. Therapeutic effects were concordant with changes in pharmacodynamic markers, including HSP90-p23 dissociation, decreases in ERBB2 and P-AKT, and increased HSP70 protein levels.

**Conclusion:**

NVP-AUY922 is a potent small molecule HSP90 inhibitor showing significant activity against breast cancer cells in cellular and *in vivo *settings. On the basis of its mechanism of action, preclinical activity profile, tolerability, and pharmaceutical properties, the compound recently has entered clinical phase I breast cancer trials.

## Introduction

Targeted therapy against an oncogenic molecule or pathway has produced promising results for various hematological malignancies and solid tumors, such as imatinib against chronic myelogenous leukemia [1], gefitinib against lung cancer [2], bevacizumab and cetuximab against colon cancer [3], and tamoxifen and trastuzumab against breast cancer [4]. However, considering the complexity of breast cancer with its multiple genetic abnormalities and resistance development against current therapies, targeting a single pathway by inhibiting the activity of one component is unlikely to be effective in the long term. Thus, identification of molecular targets that modulate multiple components of one or several signaling pathways in a nongenotoxic manner would be desired for anticancer drug discovery. For this reason, heat shock protein 90 (HSP90) has attracted considerable interest in recent years as a potential therapeutic target for the identification and development of a new generation of anticancer drugs to treat breast cancer and other malignancies [5].

HSP90 is a ubiquitously expressed molecular chaperone playing an important role in the post-translational conformational maturation and activation of a large number of client proteins that have been implicated in oncogenesis [6]. HSP90 is functional as a dimer and operates in a highly regulated ATP-fueled cycle together with a group of cochaperones (see [7] for a current overview). Inhibition of the ATPase activity at the N-terminus of HSP90 is being exploited by all inhibitors that have entered the clinic so far. Currently, the most advanced HSP90 inhibitors in clinical trials are of the benzoquinone ansamycin class, which have shown promising activity in human tumor xenograft models [6,8] and are currently undergoing phase II/III clinical trials in solid tumors and hematological malignancies. The most studied compound of this class, tanespimycin (17-AAG), has relatively poor physiochemical properties, making formulation for clinical delivery a challenge [9]. This issue has been addressed, in part, through the identification of the water-soluble analog alvespimycin (17-DMAG) [8], but the development of HSP90 inhibitors with more favorable pharmaceutical properties is being intensely pursued.

Breast cancer is a prime target indication for HSP90 inhibitors due to the relatively good understanding of the role of this chaperone in the turnover and folding of steroid hormone receptors [10-13]. The estrogen receptor (ER) antagonist tamoxifen is used as the standard of care in patients with ER-positive breast cancer [14]. However, there is medical need for alternative treatment strategies since most tumors eventually develop tamoxifen resistance even if they remain ER-positive [15]. In addition to ER, a number of other HSP90 client proteins have been shown to be involved in breast cancer progression such as those that are important for signaling through the phosphahtidylinositol-3-kinase (PI3K/p110α)/protein kinase B (PKB/AKT) pathway, including epidermal growth factor receptor (EGFR) 1 and 2 (ERBB2) and AKT [16,17]. In fact, one of the most well-defined client proteins in breast cancer is the receptor tyrosine kinase ERBB2/Her2/EGFR2. Overexpression of this protein in breast tumors leads to activation of the PI3K/AKT pathway [18]. This pathway is oncogenic in many tumors by controlling processes such as cell growth, proliferation, and generation of survival signals [19,20]. HSP90 inhibitors affect AKT activity indirectly through depletion of upstream signaling molecules (for example, ERBB family members) and directly by preventing HSP90-dependent conformational stability of AKT [17,21,22].

In this report, we demonstrate that the novel small molecule compound NVP-AUY922 potently inhibits HSP90 *in vitro*, has good pharmaceutical and pharmacological properties, and exhibits potent antitumor activity at tolerated doses in an ER- and ERBB2-positive human breast cancer model. The data provide a preclinical rationale to support phase I clinical trials with NVP-AUY922 in patients with breast cancer.

## Materials and methods

### NVP-AUY922 solution and formulation

The identification and structure of NVP-AUY922 have been described in detail elsewhere [23]. For *in vitro *experiments, stock solutions of NVP-AUY922 were prepared in 100% dimethyl sulfoxide at 10 mM and stored at -20°C. For intravenous (i.v.) administration, the free base of NVP-AUY922 was formulated in 60 mM lactic acid or 2.5% ethanol, 20% 50 mM tartaric acid, 77.5% (5% glucose in water [D5W] containing 1% Tween 80) vol/vol. An optimized NVP-AUY922 salt with high solubility in aqueous solutions was formulated in D5W for i.v. administration and delivered in a volume of 10 mL/kg.

### Established cell lines and patient-derived primary tumors

Established cell lines were obtained from the American Type Culture Collection (ATCC) (Manassas, VA, USA) and cultured in Dulbecco's modified Eagle's medium (DMEM)/F-12 supplemented with 10% fetal calf serum (FCS), with the exception of BT-474 cells, which were cultured in DMEM supplemented with 10% FCS. A clonogenic assay from primary human breast tumors was performed in a 24-well format as described [24] (Oncotest GmbH, Freiburg, Germany). Briefly, serially passaged solid human xenografts growing subcutaneously in nude mice (NMRI nu/nu strain) were disaggregated, and 4 × 10^4 ^to 8 × 10^4 ^viable cells were added to 0.2 mL of Iscove's medium (supplemented with 20% vol/vol FCS and 1% vol/vol gentamicin) containing 0.4% agar and plated on top of the base layer (0.75% agar). After 24 hours, drug was added in an additional 0.2 mL of medium and incubated at 37°C in a humidified atmosphere containing 7.5% CO_2_. At the time of maximum colony formation (8 to 20 days), counts were performed with an automatic image analysis system (OMNICON FAS IV; Bio-Sys GmbH, Karben, Germany).

### Western blot analysis and immunoprecipitation

Protein expression analysis in the panel of seven established breast cancer cell lines was assessed by Western blotting. Total cell extracts were prepared and electrophoresed in SDS-polyacrylamide gel and transferred to polyvinylidene fluoride (PVDF) membrane (Millipore Corporation, Billerica, MA, USA). The following antibodies were used for immunoblotting: Her3 (sc-285; Santa Cruz Biotechnology, Inc., Santa Cruz, CA, USA), Her2 (ab8054; Abcam, Cambridge, UK), phosphor-Her2 (Tyr1248) (44&#8211900, Invitrogen Corporation, Carlsbad, CA, USA) EGFR (#2232; Cell Signaling Technology, Inc., Danvers, MA, USA), phosphor-Akt (Ser473) (#9271; Cell Signaling Technology, Inc.), Akt (#9272, Cell Signaling Technology, Inc.), ER-α (sc-542; Santa Cruz Biotechnology, Inc.), PI3K (p110α) (#4254; Cell Signaling Technology, Inc.), PDK1 (#3062; Cell Signaling Technology, Inc.), HSP70 (SPA-810; Stressgen Bioreagents, now part of Assay Designs, Inc., Ann Arbor, MI, USA), HSP90 (SPA-845; Assay Designs, Inc.), Hsc70 (sc-7298, Santa Cruz Biotechnology, Inc.), Rb (#9309; Cell Signaling Technology, Inc.), pMEK1/2 (#9121, Cell Signaling Technology, Inc.), pERK (#9101; Cell Signaling Technology, Inc.), Bax (#2772, Cell Signaling Technology, Inc.), Bcl-2 (sc-492; Santa Cruz Biotechnology, Inc.), Bad (#9292; Cell Signaling Technology, Inc.), Bad (#9292; Cell Signaling Technology, Inc.), and Bcl-XL (#2762; Cell Signaling Technology, Inc.).

To assess total levels of AKT, phosphorylated AKT, β-tubulin, and ERBB2 in the cell extracts, 20 to 30 μg of total protein was resolved by the appropriate-percentage SDS-PAGE. The following antibodies were used for immunoblotting: anti-AKT (cat. no. 9272, rabbit polyclonal; Cell Signaling Technology, Inc.), anti-phospho-AKT (Ser473) (cat. no. 9271, rabbit polyclonal; Cell Signaling Technology, Inc.), anti-β-tubulin (cat. no. T 4026, mouse monoclonal, clone Tub2.1; Sigma-Aldrich, St. Louis, MO, USA), and anti-ERBB2 (cat. no. 28-0004, rabbit polyclonal; Zymed Laboratories Inc., now part of Invitrogen Corporation, Carlsbad, CA, USA). Bound antibodies on PVDF immunoblots were detected by Amersham ECL (Amersham, now part of GE Healthcare, Little Chalfont, Buckinghamshire, UK) or infrared fluorescence detection (LI-COR Biosciences, Lincoln, NE, USA).

For immunoprecipitation, 300 μg of total protein was immunoprecipitated with 5 μg of rat anti-HSP90α monoclonal antibody (SPA-840, clone 9D2, isotype: IgG2a; Assay Designs, Inc.). Proteins were resolved by 12% SDS-PAGE and transferred to PVDF membranes. The antibodies used were an anti-HSP90α antibody (cat. no. SPS-771, rabbit polyclonal; Assay Designs, Inc.) and an anti-p23 antibody (cat. no. ALX-804-023, mouse monoclonal, clone JJ3, isotype: IgG1; ALEXIS Corporation, Lausen, Switzerland).

### Immunohistochemistry

Formalin-fixed paraffin-embedded tissue sections were stained using the Ventana Discovery System (Ventana Medical Systems, Inc., Tucson, AZ, USA). Deparaffinization of tissue sections and heat-induced epitope retrieval using Standard Cell Conditioning Solution 1 (Ventana Medical Systems, Inc.) were performed directly on the System. A rabbit anti-human ERBB2 (HER2) monoclonal antibody (Lab Vision Corporation, Fremont, CA, USA) and a mouse anti-human HSP70 (Assay Designs, Inc.) were prepared in Dako diluent (Dako North America, Inc., Carpinteria, CA, USA) and used at concentrations of 2 ug/mL for 32 minutes and 10 ug/mL for 60 minutes, respectively. For HSP70 staining, slides were incubated with a biotin-labeled anti-mouse IgG1 (Research Diagnostics, Inc., now known as Fitzgerald Industries International, Concord, MA, USA) at a concentration of 1.25 ug/mL diluted in M.O.M. (Mouse-on-Mouse) (Vector Laboratories, Peterborough, UK). Detection using a 3,3'-diaminobenzadine reaction was performed on the section by using the Ventana OmniMAP DAB for the ERBB2 antibody and the Ventana DAB Map reagent for the HSP70 antibody (Ventana Medical Systems, Inc.). Each tissue section was subsequently counterstained with hematoxylin. To ensure antibody specificity, consecutive tissue sections were incubated with normal isotype-matched immunoglobulins (rabbit IgG; Jackson ImmunoResearch Laboratories, Inc., West Grove, PA, USA, and mouse IgG1; Lab Vision Corporation) used at concentrations equivalent to Her2 and HSP70 antibodies. Stained tissue sections were quantified using the Aperio Digital Pathology System (Aperio Technologies, Inc., Vista, CA, USA).

### Breast cancer xenograft model and efficacy studies

The ER-positive ERBB2-overexpressing cell line BT-474, which initially was derived from a human breast ductal carcinoma established from a solid invasive ductal carcinoma of the breast of a 60-year-old woman, was purchased from the ATCC (HTB-20). The cells were grown in DMEM high glucose (4.5 g/L) supplemented with 10% FCS, 200 mM l-glutamine, and 1% sodium pyruvate (BioConcept, Allschwil, Switzerland). Two or three days prior to cell inoculation, each mouse was subcutaneously implanted on the upper dorsal side with a 17β-estradiol pellet (25 μg/day, 90-day release; Innovative Research of America, Sarasota, FL, USA) using a trocar needle. BT-474 cells (5 × 10^6^) were injected in 200 μL of Matrigel/Hanks' balanced salt solution (1:1 vol) (BD Matrigel™ Basement Membrane Matrix; BD Biosciences, San Jose, CA, USA) subcutaneously in the right flank. Invasive procedures were performed under Forene anesthesia. All experiments were performed using female Harlan HsdNpa: Athymic Nude-nu mice that were obtained from Novartis internal breeding stocks (Laboratory Animal Services, Novartis Pharma AG, Basel, Switzerland). The animals were kept under optimized hygienic conditions with 12-hour dark/12-hour light conditions. The animals were fed food and water *ad libitum*. All animal experiments were performed in strict adherence to the Swiss law for animal protection. The experimental protocols were approved by the Swiss Cantonal Veterinary Office of Basel-Stadt.

### Tumor volume measurements

Treatment with NVP-AUY922 was initiated when the average tumor volume reached approximately 100 mm^3^. Tumor growth and body weights were monitored at regular intervals. The xenograft tumor sizes were measured manually with calipers, and the tumor volume was estimated using the formula (w × l × h × π/6), where width (w), height (h), and length (l) are the three largest diameters [25].

### Pharmacokinetic analysis

Female athymic BT-474 tumor-bearing mice with tumors of approximately 250 mm^3 ^received an i.v. dose of 30 mg/kg of NVP-AUY922. At various time points, mice (n = 4) were sacrificed and blood and tissues (tumor, liver, lung, heart, and muscles) were dissected. Concentrations of NVP-AUY922 in plasma and tissues were determined by high-pressure liquid chromatography/tandem mass spectrometry (HPLC/MS-MS) operated in electrospray ionization-positive mode. Frozen tissues were minced, then homogenized in an equal volume of ice-cold phosphate-buffered saline (Sigma P4417; Sigma-Aldrich) using a Polytron homogenizer (TP18-10; IKA, Staufen, Germany) and keeping the material cold during the homogenization. After the addition of 50 μL of internal standard (1 μg/mL) to analytical aliquots (25 to 250 μL) of plasma or tissue homogenate, the proteins were precipitated by the addition of an equal volume of acetonitrile and processed further for chromatographic separation. After three repetitions of protein precipitation by the addition of an equal volume of acetonitrile followed always by evaporation to dryness, the samples were redissolved in 100 μL of acetonitrile/water (1/9 vol/vol) containing 0.2% vol/vol formic acid. An aliquot (5 μL) of this solution was separated on a RESECT™ Ultra Cyano reverse-phase HPLC column (column size 50 × 1 mm, particle size 3 μm, preceded by a guard column: Phenomenex™ AJO-4304 Phenylpropyl, size 4 × 2 mm (Phenomenex, Torrance, CA, USA)) with a mobile phase consisting of a mixture of 0.2% formic acid in water (solvent A) and 0.2% formic acid in acetonitrile (solvent B). The column eluent was introduced directly into the ion source of the triple-quadrupole mass spectrometer Quattro Ultima™ (Micromass Limited, now part of Waters Corporation, Milford, MA, USA) controlled by Masslynx™ 4.0 software. Positive electrospray ionization multiple-reaction monitoring was used for the MS/MS detection of the analyte. Precursors to product ion transitions of m/z 466.35 → m/z 308.20 for NVP-AUY922-NX and m/z 480.40 → m/z 308.15 for IS VER814 were used. The limits of quantification were set to 4 ng/mL and 10 ng/g for plasma and tissues, respectively (coefficient of variation and overall bias less than 30%). Regression analysis and further calculations were performed using QuanLynx™ 4.0 (Waters Corporation, Milford, MA, USA) and Excel™ 2002 (Microsoft Corporation, Redmond, WA, USA). Concentrations of unknown samples were calculated from the peak area ratio of the product ion of the analytes to the product ion of its internal standard (ordinate) against the nominal concentration (abscissa). Assay linearity was indicated by an overall regression coefficient of 0.9975.

### Statistical analysis

When applicable, results are presented as mean ± standard error of the mean. Tumor and body weight data were analyzed by analysis of variance (ANOVA) with the *post hoc *Dunnett test for comparison of treatment versus control groups. The *post hoc *Tukey test was used for intragroup comparison. Statistical analysis was performed using GraphPad Prism 5 (GraphPad Software, Inc., San Diego, CA, USA). As a measure of efficacy, the %T/C value is calculated at the end of the experiment according to (Δtumor volume_treated_/Δtumor volume_control_) × 100, where Δtumor volumes represent the mean tumor volume on the evaluation day minus the mean tumor volume at the start of the experiment.

## Results and Discussion

### NVP-AUY922 is a potent inhibitor of breast cancer cell proliferation *in vitro*

**Figure 1:**
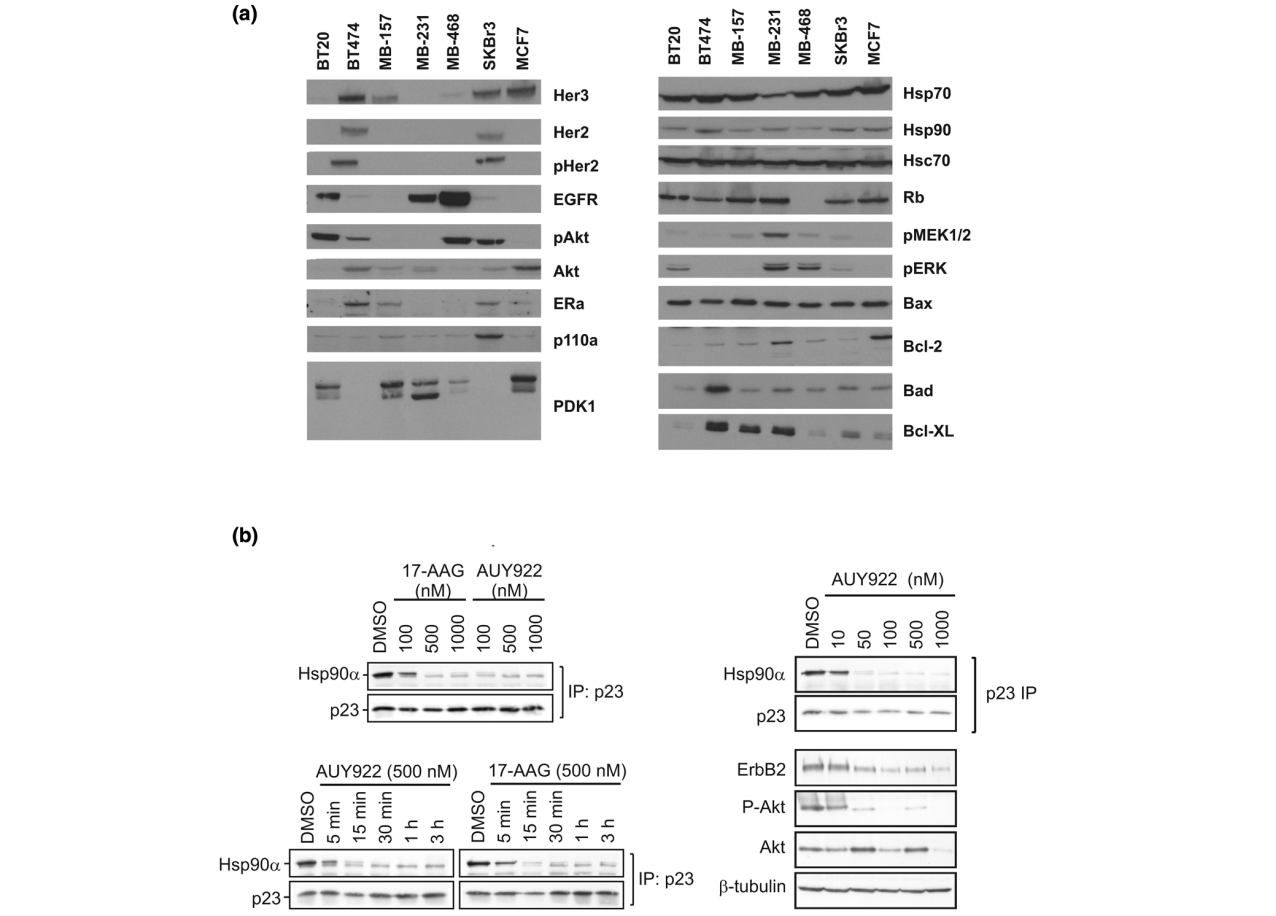
Protein expression analysis in a panel of human breast cancer cell lines and effect of NVP-AUY922 on the HSP90-p23 complex in BT-474 cells. **(a) **The expression of HSP90 and specific proteins affected by HSP90 inhibition (Her3 [EGFR3], Her2 [ErbB2, EGFR2], phospho Her2 [pHer2], EGFR, AKT [PKB], phospho-AKT [pAkt], estrogen receptor-alpha [ERa], PI3K [p110α], PDK1, HSP70, Hsc70, Rb, pMEK1/2, pERK, Bax, Bcl-2, Bad, and Bcl-XL) was analyzed in seven human breast cancer cell lines (BT20, BT-474, MDA-MB-157 [MB-157], MDA-MB-231 [MB-231], MDA-MB-468 [MB-468], SKBr3, and MCF-7) by Western blot analysis. **(b) **The ERBB2-overexpressing and estrogen receptor-positive cell line BT-474 was chosen for studies of the kinetics and concentration-dependent dissociation of HSP90-p23 complexes and client proteins in the presence of NVP-AUY922 or tanespimycin (17-AAG). The amount of p23 associated with HSP90 was determined by immunoprecipitating p23 followed by immunoblotting for HSP90. The levels of ERBB2, AKT, phosphorylated AKT and β-tubulin were determined by immunoblotting. DMSO, dimethyl sulfoxide; HSP90, heat shock protein 90.

We have previously shown NVP-AUY922 to be a potent selective inhibitor of HSP90. In competitive fluorescence polarization assays, NVP-AUY922 inhibited HSP90α and HSP90β with similar IC_50 _(median inhibition concentration) values of 13 and 21 nM [23,26], respectively. In a representative panel of human tumor cell lines (including prostate, breast, ovarian, colon, lung, melanoma, and glioblastoma), NVP-AUY922 inhibited cell proliferation with low nanomolar potency; GI_50 _(the concentration that inhibits cell growth by 50%) values were in the range of 2.3 to 50 nM [23]. To better elucidate the clinical utility of NVP-AUY922, we investigated the effects of the compound against a panel of human breast cancer cell lines. NVP-AUY922 was potent against six of the seven breast cancer cell lines tested, inhibiting *in vitro *growth with an average GI_50 _value of 5.4 nM (Table 1). When compared directly with 17-AAG, NVP-AUY922 was between 3.6- and 300-fold more active at inhibiting tumor cell growth. MDA-MB-157 exhibited around a 14-fold reduced sensitivity to NVP-AUY922 compared with the next most insensitive cell line. In an attempt to understand the differential cellular response of HSP90 inhibition by NVP-AUY922, the expression levels of HSP90, HSP70, various client proteins (EGFR1/Her1, ERBB2, AKT, and ER), and those involved in apoptotic responses (Bax, Bad, and Bcl-XL) were determined (Figure 1a). No clear correlation between protein expression or phosphorylation status and cell line response could be ascertained. More plain was the heterogeneity in pathway composition between the various cell lines. BT-474 and SKBr3 have often been used to examine the effects of HSP90 inhibitors in high ERBB2 expressing breast cancer cell lines. However, they differ quite markedly in the expression of other factors (such as p110α) central to this pathway. Therefore, attempting to define sensitive clinical populations via the expression of a single client protein or a limited number of client proteins may not be a suitable approach, in particular when a substantial number of proteins can be affected by HSP90 modulation. To further evaluate the efficacy of NVP-AUY922 against human breast tumors, the ability of NVP-AUY922 to inhibit the colony-forming potential of primary tumor stem cells growing in soft agar was examined [24]. Tumor cells were derived from patient samples directly transplanted to and subsequently serially passaged in nude mice. This clonogenic assay system may better reflect the *in vivo *situation than *in vitro *assays using established tumor cell lines and has been shown to be predictive for further *in vivo *evaluation of anticancer drugs [27]. NVP-AUY922 was highly effective at inhibiting the growth of five out of the six human breast cancer explants tested, with average GI_50 _and GI_70 _(the concentration that inhibits cell growth by 70%) values of 191 and 379 nM, respectively (Table 2). One of the tumor explants was highly resistant to NVP-AUY922 treatment, exhibiting a GI_50 _value of greater than 10 μM (the highest concentration evaluated).

**Table 1:** Antiproliferative effect of NVP-AUY922 against a panel of human breast cancer cell lines

Cell line	ErbB2/ER status	NVP-AUY922	17-AAG
		
		nM	
BT-474	ErbB2^+^/ER^+^	3.1 ± 1.4	17.4 ± 5.2
BT20	ErbB2^-^/ER^-^	4.0 ± 1.4	18.9 ± 4.8
MDA-MB-157	ErbB2^-^/ER^-^	126 ± 37	29.5 ± 20.8
MDA-MB-231	ErbB2^-^/ER^-^	7.0 ± 1.7	2,057 ± 571
MDA-MB-468	ErbB2^-^/ER^-^	6.3 ± 2.6	1,657 ± 390
SkBr3	ErbB2^+^/ER^-^	3.3 ± 0.9	11.9 ± 8.0
MCF-7	ErbB2^-^/ER^-^	8.8 ± 1.8	69.0 ± 18.3

The concentrations of NVP-AUY922 and tanespimycin (17-AAG) that inhibit cell number by 50% (GI_50_) were determined. ErbB2 and estrogen receptor (ER) status is indicated.

**Table 2:** Antiproliferative effect of NVP-AUY922 against primary breast cancer

Cell line	IC_50_	IC_70_
	
	nM	
MAXF 1162	304	505
MAXF 1322	29	48
MAXF 1384	209	477
MAXF 401	78	295
MAXF 574	>10,000	>10,000
MAXF 583	333	569

The concentrations of NVP-AUY922 that inhibit colony formation by 50% (IC_50_) or 70% (IC_70_) were determined (Oncotest GmbH, Freiburg, Germany) [24].

### NVP-AUY922 induces HSP90-p23 dissociation and client protein depletion in a concentration- and time-dependent manner

The classic method of following the cellular activity of HSP90 inhibitors is through the proteasome-dependent degradation of HSP90 client proteins such as ERBB2 and AKT and the concomitant loss of signaling through the affected pathways as determined by, for example, decreased AKT phosphorylation. This method, however, does not directly demonstrate that the catalytic activity of HSP90 has been blocked. An alternative method of studying the cellular effects of HSP90 inhibitors, which more closely monitors the catalytic cycle of HSP90, is through the evaluation of disruption of the HSP90-p23 complex [28]. HSP90 recruits various cochaperones at different points in its catalytic cycle, and the association of HSP90 with p23 is essential for HSP90 activity and client protein stability. HSP90-p23 interaction requires ATP binding but not ATP hydrolysis in biochemical assays, and p23 specifically recognizes HSP90-ATP complexes but not HSP90 alone [28]. Thus, treatment with the ATP competitive inhibitor 17-AAG results in the dissociation of the HSP90-p23 complex, a feature that recently has been exploited in human cell lines and xenograft using a split HSP90-p23 Renilla luciferase protein fragment-assisted complementation assay [29]. Destabilization of the HSP90-p23 interaction in tumor cells and the subsequent measurement using immunoprecipitation therefore can be used to monitor the effect of HSP90 inhibitors on the HSP90 catalytic cycle (Figure 1b). In BT-474 cells, NVP-AUY922 and 17-AAG caused a concentration-dependent decrease in the amount of HSP90α co-immunoprecipitating with p23. NVP-AUY922 appears to be more potent than 17-AAG at inhibiting the HSP90-p23 interaction. This observation is in accordance with the increased cellular growth inhibition potency obtained with NVP-AUY922 in comparison with 17-AAG (Table 1). The inhibition of the HSP90-p23 association occurs rapidly following addition of both compounds. Specifically, NVP-AUY922 causes HSP90-p23 complex dissociation to happen within 5 minutes and complete effect to happen within 15 minutes. For 17-AAG, the time to onset of complex destabilization is slightly delayed compared with NVP-AUY922 but full inhibition still occurs within 15 minutes (Figure 1b). To confirm that complex destabilization was affecting client protein status, we correlated complex dissociation by immunoprecipitation with client protein degradation. Treatment of BT-474 cells with 50 nM NVP-AUY922 caused a clear dissociation between p23 and HSP90α, resulting in the loss of HSP90α from p23 immunoprecipitates (Figure 1b). This clearly coincided with a decrease in total ERBB2 levels and subsequent inhibition of downstream signaling as measured by a decrease in the levels of phospho-AKT. Under these experimental conditions, significant reduction of protein AKT levels requires a higher NVP-AUY922 concentration of around 1 μM.

### Biodistribution measurements

**Figure 2:**
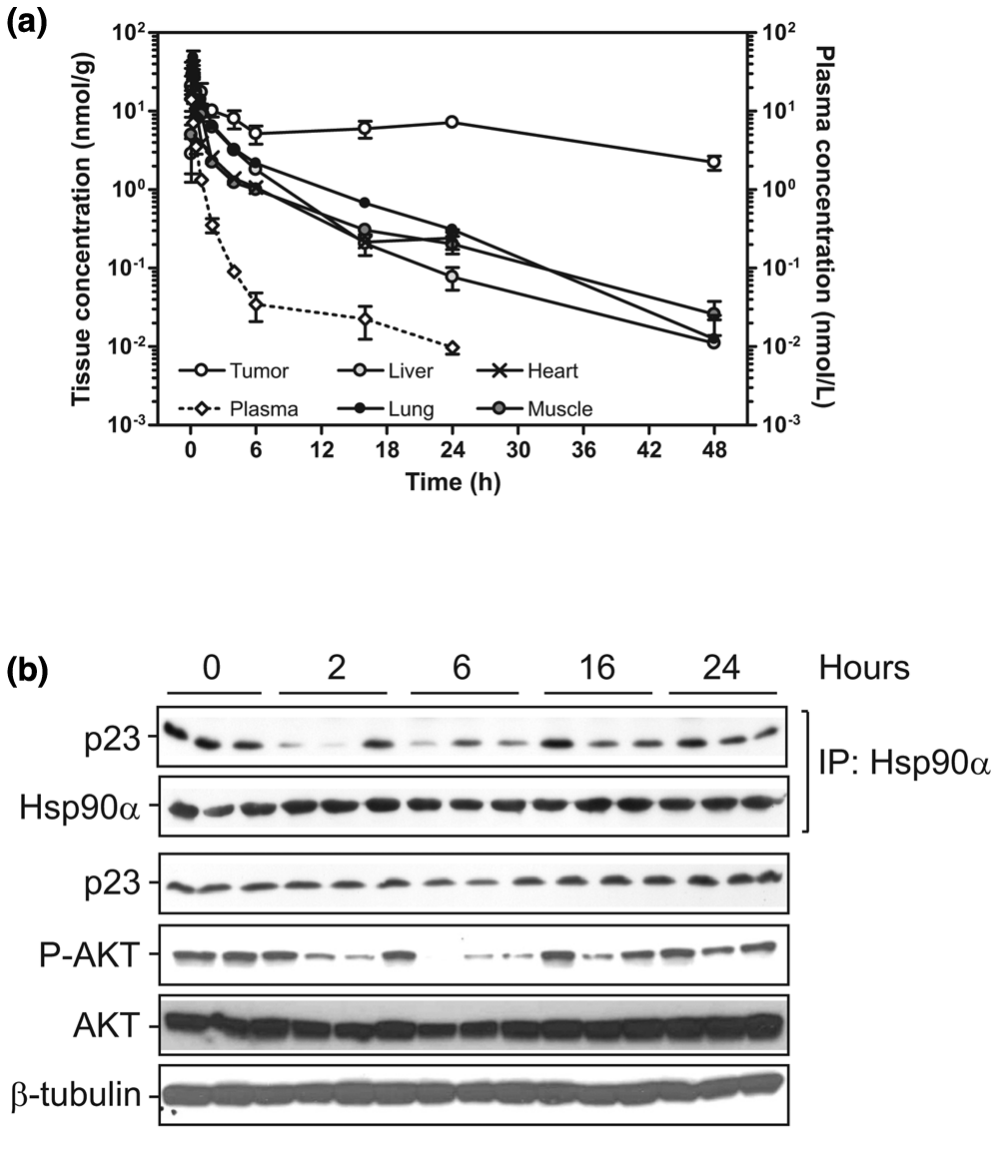
Pharmacokinetic/pharmacodynamic analysis of NVP-AUY922 in BT-474 tumor-bearing nude mice. A pharmacokinetic profile of NVP-AUY922 in BT-474 tumor xenografts, plasma, and organs (liver, heart, lung, and muscle) after administration of a single dose is shown. Female athymic mice bearing subcutaneous xenotransplants of the human ductal breast carcinoma BT-474 of approximately 250 mm^3 ^received a single dose of 30 mg/kg of NVP-AUY922 (intravenous) at 0 hours. **(a) **NVP-AUY922 concentrations were determined by HPLC/MS-MS analysis using an internal standard method. The limits of quantification for plasma and tissues were set to 4.0 ng/mL and 10 ng/g, respectively. Bars represent standard error of the mean (n = 4). **(b) **Pharmacodynamic analysis in BT-474 xenografts following a single NVP-AUY922 dose. The amount of p23 associated with HSP90 was determined by immunoprecipitating HSP90 followed by immunoblotting for p23. The levels of AKT, phosphorylated AKT and β-tubulin were determined by immunoblotting. HPLC/MS-MS, high-pressure liquid chromatography/tandem mass spectrometry; HSP90, heat shock protein 90; IP, immunoprecipitation.

Since NVP-AUY922 exhibited a strong antiproliferative effect against established and primary breast cancer cells (Tables 1 and 2), we evaluated the properties of the compound *in vivo*. To this end, a breast cancer xenograft model based on the BT-474 cell line was established. The antitumor effect of 17-AAG has been thoroughly evaluated in this model, which is known to be highly sensitive to HSP90 inhibitors when grown as subcutaneous (s.c.) xenograft tumors [21,30]. This cell line is expressing high levels of ERBB2 and ER-α (Figure 1a) and depends on exogenous estrogen supplement for growth as s.c. xenografts in athymic nude mice. The biodistribution and pharmacokinetic profile of NVP-AUY922 was determined by HPLC/MS-MS (Figure 2a). The pharmacokinetic parameters derived from these data are summarized in Table 3. We found that NVP-AUY922 is rapidly distributed from the bloodstream into tissues and has a short half-life in plasma. Plasma clearance was 8.9 L/hour and a large volume of distribution of 18.9 L was determined. The pharmacokinetic profile of NVP-AUY922 is characterized by a biphasic decline in plasma. Plasma concentrations were below levels of quantitation 48 hours after compound administration. The apparent terminal elimination half-life for NVP-AUY922 in plasma was 10 hours based on the last three time points. The dose-normalized plasma exposure following i.v. dosing at 20 mg/kg resulted in a similar value as with 30 mg/kg (0.28 versus 0.24 hours* μmol/L), indicating dose linearity within this dose range (data not shown). The tissue exposures over the course of 48 hours were 5*fold (muscle and heart), 7*fold (liver), and 10*fold (lung) greater than in plasma. The terminal elimination half-life from the analyzed non-tumor tissues was between 5.5 and 8 hours. Importantly, the tumor exposure was approximately 47*fold higher than that in plasma, maintaining a concentration of 2.22 nmol/g 48 hours after compound administration. This drug level corresponded to approximately 13% of the C_max _(highest concentration) (16.3 nmol/g) and resulted in a terminal half-life of 25 to 30 hours. Interestingly, retention in tumor xenografts was also observed for 17*AAG [31], indicating that this may be a target-related phenomenon.

**Table 3:** NVP-AUY922 biodistribution: pharmacokinetic parameters

	Plasma	Tumor	Liver	Muscle	Heart	Lung
C_max_, μmol/L or nmol/g	14.08 ± 4.14	16.36 ± 3.84	30.94 ± 5.58	27.45 ± 6.64	36.86 ± 7.42	48.48 ± 10.11
T 1/2 _elimination λ_z_, hours	10.3	30	7.7	8	5.6	5.5
AUC_(0-8)_, hours* μmol/L or hours*nmol/g	7.3	345	53	35	32	71

The concentration of NVP-AUY922 was determined by high-pressure liquid chromatography/tandem mass spectrometry after a single intravenous administration of 30 mg/kg to BT-474 tumor-bearing mice. The highest concentration (C_max_), terminal half-life (T 1/2 _elimination λ_z_), and exposure as determined by the area under the curve (AUC) were determined for each organ, plasma, and BT-474 xenograft (tumor).

### NVP-AUY922 affects pharmacodynamic markers *in vivo*

**Figure 3:**
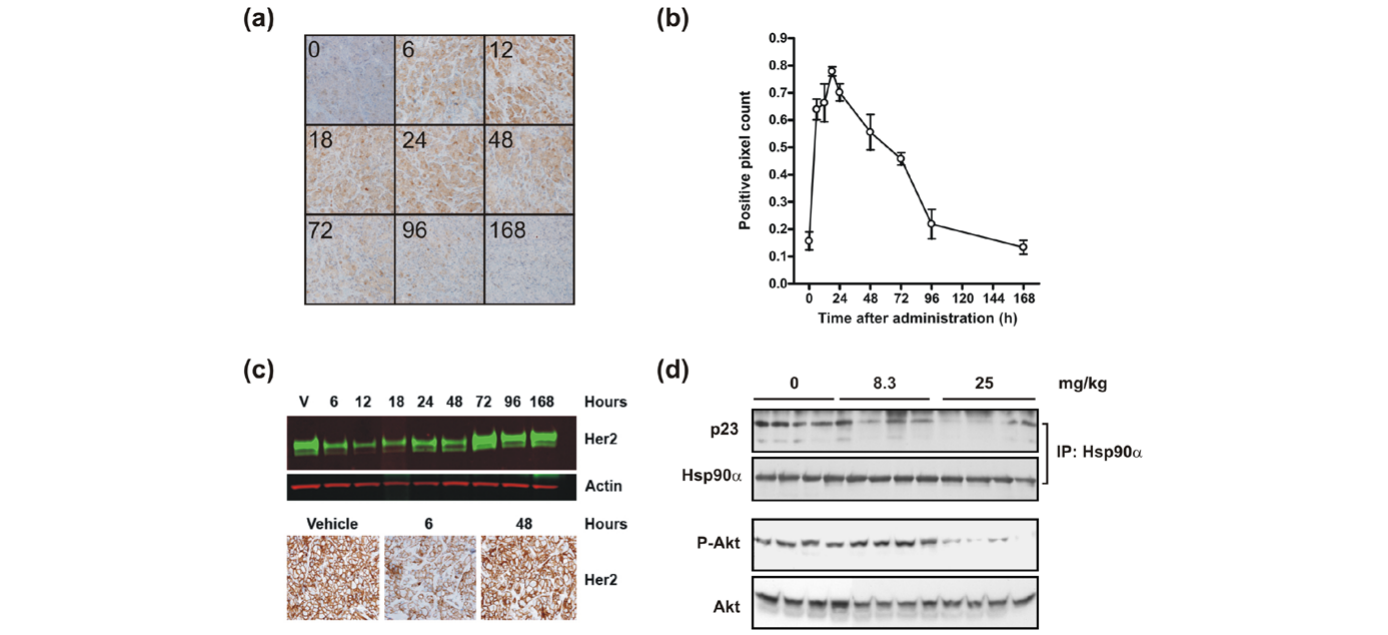
Analysis of the kinetics of HSP70 induction and ERBB2 degradation following a singe dose of NVP-AUY922. BT-474 tumor-bearing animals were administered 50 mg/kg NVP-AUY922 at 0 hours. **(a) **The protein levels of HSP70 were determined by immunohistochemistry during the following week. **(b) **The quantification of the protein levels of HSP70. **(c) **ERBB2 protein levels were determined by immunohistochemistry and Western blot analysis. **(d) **The lowest single dose of NVP-AUY922 which mediates HSP90-p23 dissociation and reduced AKT phosphorylation was determined by immunoprecipitation and Western blot analysis. HSP70, heat shock protein 70; HSP90, heat shock protein 90.

The biodistribution and pharmacokinetic profile of NVP-AUY922 in the BT-474 xenograft model encouraged us to assess whether NVP-AUY922 is capable of directly interfering with the catalytic cycle of HSP90 with concomitant depletion of HSP90 client proteins *in vivo *(Figure 2b). To asses whether systemic administration of NVP-AUY922 could affect the association between p23 and HSP90 observed *in vitro*, HSP90α was immunoprecipitated and co-immunoprecipitating p23 was detected by Western blotting. A significant effect of NVP-AUY922 on HSP90-p23 complex dissociation was observed at the 2- and 6-hour time points. From 16 and 24 hours after compound administration, HSP90-p23 complexes reassembled in the BT-474 xenografts. To asses whether the effect of AKT phosphorylation on Ser473 observed on cell lines grown in culture could be observed in the xenograft model, Western blot analysis was performed. We observed that the inhibition of HSP90 by a single dose of NVP-AUY922 was paralleled by reductions in phospho-AKT levels. Thus, NVP-AUY922 inhibits the catalytic cycle of HSP90 and reduces AKT signaling in xenograft tumor tissues, which is similar to the effects observed *in vitro*. Another hallmark of HSP90 inhibition is the induction of a heat shock response, which was evaluated by determining the HSP70 protein levels by immunohistochemistry (Figure 3). In this experiment, a single 50 mg/kg dose of NVP-AUY922 was administered to BT-474 xenograft-bearing mice and tumor sections were prepared at selected time points over the following week (Figure 3a). The staining intensity was quantified using automated imaging analysis (Figure 3b) and already demonstrated strongly increased HSP70 staining 6 hours after compound administration. The staining intensity peaked at the 24-hour time point and gradually returned to baseline over one week (Figure 3b). These data demonstrated a delayed heat shock response compared with the early response observed on the HSP90-p23 complex and AKT phosphorylation. Next, we determined the expression levels of the client protein ERBB2 in the same experiment by immunohistochemistry and Western blot analysis (Figure 3c). ERBB2 levels were reduced from 6 to 18 hours after administration of NVP-AUY922 but returned to baseline between 24 and 48 hours after the administration. Overall, the data suggest that a cascade of timely events was initiated in which inhibition of HSP90 ATPase activity leads to immediate HSP90-p23 dissociation followed by inhibition of downstream signaling as detected by decreased phosphorylation of AKT and degradation of client proteins (for example, ERBB2) concomitant with release of HSF-1, causing transcriptional induction of HSP70.

### Lowest dose that elicits pharmacodynamic response in tumors

The lowest dose of NVP-AUY922 which could elicit a detectable response on HSP90-p23 complexes as well as AKT phosphorylation was determined. The effect of NVP-AUY922 on selected pharmacodynamic (PD) markers was assessed by administering the drug intravenously to s.c. BT-474 tumor-bearing mice. A single dose of 8.3 or 25 mg/kg was administered and tumors were dissected 6 hours later since HSP90-p23 dissociation and AKT phosphorylation are easily detectable at this time point (Figure 2b). A dose of 25 mg/kg NVP-AUY922 caused significant disruption of HSP90-p23 complexes, whereas the 8.3 mg/kg dose caused only a subtle disruption (Figure 3d). Only the highest dose of 25 mg/kg NVP-AUY922 elicited a reduction in phospho-AKT levels. Thus, even though 8 mg/kg NVP-AUY922 appeared to cause a slight reduction in HSP90-p23 complexes, the remaining complexes are likely sufficient to maintain downstream signaling as evidenced by AKT phosphorylation.

### NVP-AUY922 exhibits potent antitumor activity at well-tolerated dose levels against an ERBB2-overexpressing ER-positive breast cancer xenograft

**Figure 4:**
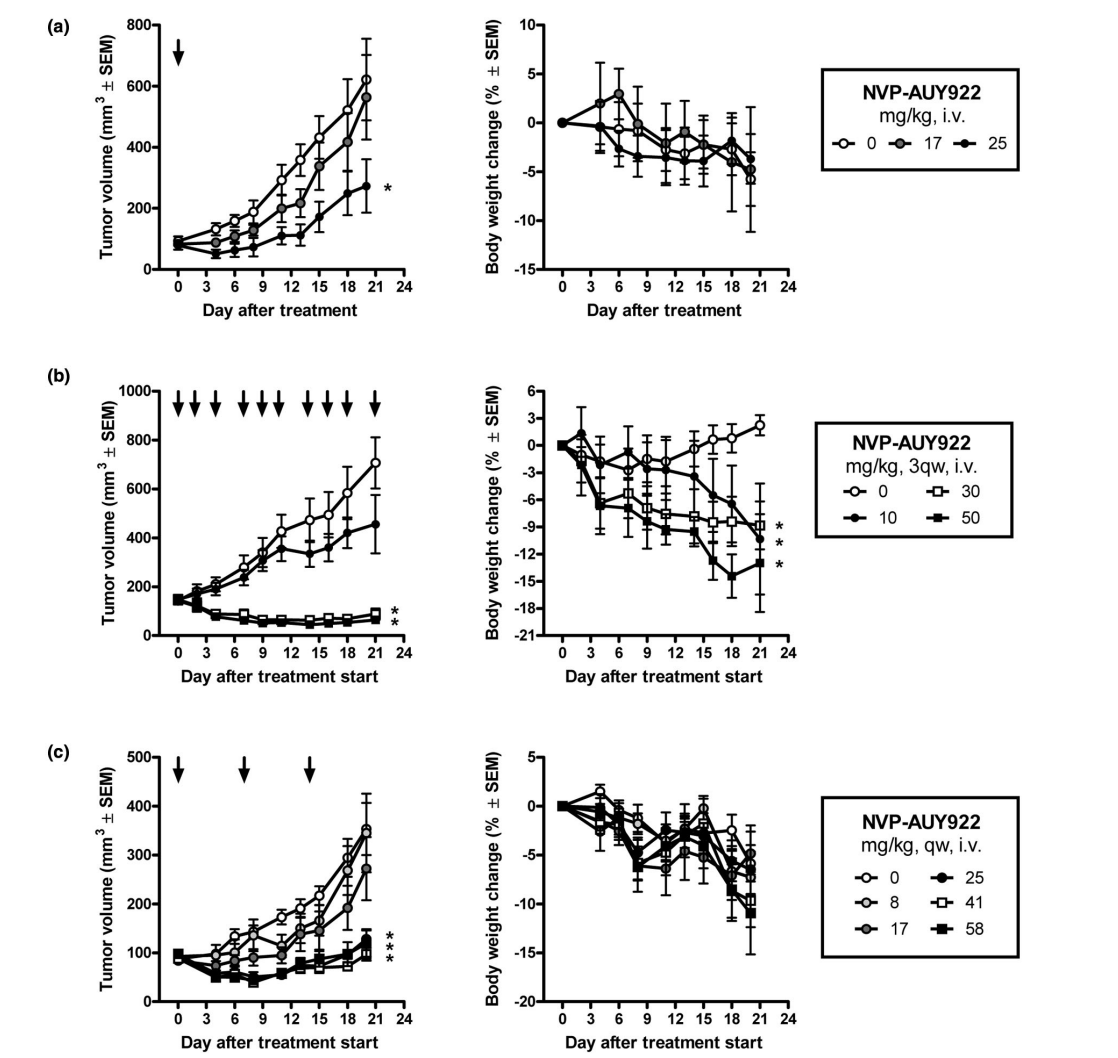
Antitumor effect and tolerability of NVP-AUY922 in a BT-474 human breast cancer xenograft model. BT-474 cells were inoculated subcutaneously in female nude mice carrying an estrogen-release pellet. When the tumors reached 100 to 200 mm^3^, drug treatment was initiated. Each group consisted of eight animals. NVP-AUY922 was administered **(a) **as a single injection, **(b) **three times per week (3qw), or **(c) **once per week (qw) at the indicated dose levels. Tumor volumes and body weights were measured three times per week. Each point represents the mean ± standard error of the mean (SEM). Arrows in right panels indicate NVP-AUY922 treatment days. Asterisks indicate statistical significance compared with vehicle-treated controls (*P *< 0.05, one-way analysis of variance *post hoc *Dunnett). i.v., intravenous.

During the drug discovery selection process for the optimal development candidate, NVP-AUY922 was administered intraperitonealy once per day to HCT16 colon tumor xenograft-bearing mice at a dose of 50 mg/kg [23]. Apart from demonstrating the antitumor potential of the compound, this study demonstrated that NVP-AUY922 did not cause significant body weight changes when administered intraperitonealy at dose levels of up to 50 mg/kg daily [23]. We wanted to elaborate on these initial findings by investigating whether the effect observed on the PD markers correlated with an antitumor effect at well-tolerated doses and regimens in the BT-474 tumor model. During the establishment of the BT-474 model, we noticed that untreated tumor- and estrogen pellet-bearing mice generally either lost or gained minimum body weight during the experiment (data not shown and Figure 4, right panels). The body weight loss is likely to make the animals more sensitive to the potential additional toxicity caused by the treatment. As such, this xenograft model is ideally suited for use to optimize the dosing regimen that gives a good efficacy/tolerability balance. To more closely simulate the administration route to be used in clinical trials, the administration schedule was optimized using i.v. administration of NVP-AUY922 (Figure 4). In the first experiment, a single dose of NVP-AUY922 was administered at around the lowest dose levels that elicited a reliable PD marker response (Figure 3d). Either 17 or 25 mg/kg was administered and tumor growth was monitored for 21 days (Figure 4a). A single administration at these dose levels did not affect body weights compared with vehicle. However, at the 25 mg/kg dose level, significant tumor growth was not observed for about 1 week after the administration. Also, at a dose level of 17 mg/kg, an indication of retarded tumor growth was observed (Figure 4a). Interestingly, at the end of the experiment, the animals in the group treated with 25 mg/kg had a tumor size corresponding to the tumor size in control animals approximately 10 days earlier and were statistically significantly smaller in this group compared with the control group at the end of the experiment (*P *< 0.05, one-way ANOVA *post hoc *Dunnett versus control). These results combined with PD data showing a response lasting more than 24 hours clearly demonstrated a likelihood of achieving good antitumor efficacy when administering NVP-AUY922 in intermittent dose regimens. To evaluate this hypothesis, we administered NVP-AUY922 three times per week (Monday, Wednesday, and Friday) at dose levels of 10, 30, and 50 mg/kg (Figure 4b). Indeed, we achieved tumor regression at the two highest dose levels whereas treatment at 10 mg/kg, which did not elicit a reproducible effect on PD markers (Figure 3d), did not result in a significant effect on tumor growth. Although these data were highly encouraging, this treatment regimen resulted in a significant body weight loss at all dose levels (Figure 4b, right panel). To further improve the administration regimen, we evaluated whether extending the recovery period in between dosing would improve tolerability without affecting antitumor effect. Since tumor growth was blocked for about 1 week after a single administration of NVP-AUY922 (Figure 4a), a once-a-week dosing regimen was investigated. Groups of eight BT-474 tumor-bearing animals were treated at dose levels of 8.3, 17, 25, 41, or 58 mg/kg. In this setting, NVP-AUY922 was highly efficacious when a dose level of 25 to 58 mg/kg was administered once per week (Figure 4c and Table 4). Dose levels of 8.3 to 17 mg/kg did not result in a significant effect on tumor growth compared with vehicle-treated control animals, correlating with the data demonstrating that to observe a consistent PD response a single dose of 25 mg/kg is needed (Figure 3d). Overall, we demonstrate that NVP-AUY922 has good pharmacokinetic properties and is efficacious and well tolerated when administered as a single agent once per week.

**Table 4:** Antitumor effect and tolerability of NVP-AUY922 against BT-474 tumor-bearing nude mice when administered once per week

	Tumor response		Host response		
	
NVP-AUY922 dose, mg/kg	T/C, percentage	Tumor volume change, cubic millimeters	Body weight change, grams	Body weight change, percentage	Survival, percentage
0	100	266 ± 51	-1.6 ± 0.9	-5.9 ± 3.2	100
8.3	95	252 ± 73	-2.2 ± 0.8	-7.3 ± 2.6	100
17	72	191 ± 61	-1.2 ± 0.7	-4.8 ± 2.9	88
25	12	32 ± 15^a^	-1.7 ± 0.6	-6.5 ± 2.5	100
41	3	9 ± 13^a^	-2.4 ± 0.6	-9.7 ± 2.7	100
58	8	22 ± 26^a^	-3.0 ± 1.2	-10.9 ± 4.2	100

BT-474 cells were inoculated subcutaneously in female nude mice carrying an estrogen-release pellet. When the tumors reached 100 to 200 mm^3^, drug treatment was initiated. Each group consisted of eight animals. NVP-AUY922 was administered once per week at the indicated dose levels. Tumor volumes and body weights were measured three times per week, and the experiment was evaluated 20 days after the first dose was administered. The percentage T/C represent the mean change in tumor volume of the treated group divided by the mean change in tumor volume of the vehicle treated control group. ^a^Statistical significance compared with vehicle-treated controls (*P *< 0.05, one-way analysis of variance *post hoc *Dunnett).

## Conclusion

HSP90 has become an increasingly attractive target for anticancer therapy [6]. HSP90 inhibitors cause pleiotropic effects through degradation of a large number of client proteins as well as induction of a heat shock response [32]. The simultaneous effect on multiple oncogenic pathways is predicted to be beneficial in a broad spectrum of cancer types that are driven by HSP90 client proteins. In this report, we focused on provided preclinical data to support the use of NVP-AUY922 for the treatment of breast cancer, an indication that is currently being pursued in clinical trials. To this end, we demonstrate that NVP-AUY922 exhibited significant antiproliferative properties against most established and primary breast cancer cells. A few breast cancer cell lines were found to be less sensitive, which may be due to drug transporters or metabolic activity. This is currently being investigated further.

The expression of a number of client proteins, signaling molecules, heat shock response proteins, and proteins involved in apoptotic pathways was determined. However, no clear correlation between a specific pathway and antiproliferative effect could be found. The majority of the work presented in this report was performed in the BT-474 breast cancer cell line, which is driven by ERBB2 and depends on estrogen for growth. However, although ERBB2 is an excellent client protein, ERBB2-negative tumors may well be driven by susceptible client proteins and respond to HSP90 inhibitors. Based on the antiproliferative profile in breast cancer cell lines, it is difficult to link NVP-AUY922 antitumor activity with a specific genetic marker such as ERBB2 expression or ER status. This makes patient stratification exceedingly difficult, as discussed elsewhere [33].

NVP-AUY922 binds very potently to HSP90 in a competitive biochemical assay [23,26]. The high biochemical potency correlated with the ability of NVP-AUY922 to directly interfere with the HSP90-p23 complex both in BT-474 cells grown in culture and as xenografts. However, for complete dissociation of the HSP90-p23 complexes within 3 hours, a concentration of approximately 15 times the BT-474 GI_50 _value (determined after continuous exposure for 2 days) is needed. These data suggest that complete inhibition of HSP90 is not needed to achieve antiproliferative effect by continuous exposure. However, short-term exposure at high drug concentrations may cause potent HSP90 inhibition in cells that are highly dependent on HSP90, such as tumors [34]. The short-term exposure causes the initiation of downstream events (for example, client protein degradation) from which affected tumor cells recover slowly. This hypothesis supports intermittent dosing of HSP90 inhibitors. When BT-474 tumor-bearing animals were administered NVP-AUY922 weekly at doses that cause tumor concentrations to reach 1,000 times the GI_50 _value for approximately 2 days, significant tumor growth was not observed for about 1 week. In addition, the weekly schedule was well tolerated. Therefore, an NVP-AUY922 intermittent dosing regimen gives optimal tolerability without significantly affecting efficacy. This has also been found to be the case for other HSP90 inhibitors such as 17-AAG, which is being administered in an intermittent dose regimen [35]. Overall, our data offer further support for NVP-AUY922 to enter clinical trials.

## Abbreviations

17-AAG = tanespimycin; ANOVA = analysis of variance; ATCC = American Type Culture Collection; D5W = 5% glucose in water; DMEM = Dulbecco's modified Eagle's medium; EGFR = epidermal growth factor receptor; ER = estrogen receptor; FCS = fetal calf serum; GI_50 _= the concentration that inhibits cell growth by 50%; HPLC = high-pressure liquid chromatography; HSP90 = heat shock protein 90; i.v. = intravenous; MS-MS = tandem mass spectrometry; PD = pharmacodynamic; PI3K = phosphahtidylinositol-3-kinase; PVDF = polyvinylidene fluoride; s.c. = subcutaneous.

## Competing interests

MRJ, JS, TR, CTG, JB, CQ, AB, RC, CG-E, and PC are employees and stockholders of Novartis Pharma AG. AM and MJD are employees and stockholders of Vernalis, Plc.

## Authors' contributions

MRJ participated in the design and coordination of the studies, conducted *in vivo *pharmacology experiments, and is the author responsible for the manuscript. JS participated in study design and coordination. TR developed and conducted the HSP90-p23 binding assay. AM conducted cellular proliferation assays and PD analysis. CTG conducted immunohistochemistry and Western blot analysis. JB conducted the pharmacokinetic studies. CQ gave clinical advice. AB coordinated immunohistochemistry studies. RC coordinated *in vivo *studies. MJD and CG-E coordinated studies. PC was responsible for overall study design and coordination. All authors participated in discussions and read and approved the final manuscript.
